# Randomized comparison of gamified mobile app–based training versus conventional learning for pneumothorax detection in chest radiographs

**DOI:** 10.1186/s12909-026-09167-x

**Published:** 2026-04-13

**Authors:** Jacob Jalil Hassan, Christian Boschenriedter, Arian Taheri Amin, Silvio Wermelskirchen, Gunther Hempel, Timm Denecke, Hans-Jonas Meyer

**Affiliations:** 1 Department of Diagnostic and Interventional Radiology, University Hospital Leipzig, Leipzig, Germany; 2 Department of Diagnostic and Interventional Radiology, Medical Faculty and University Hospital Düsseldorf, Heinrich-Heine- University Düsseldorf, Düsseldorf, Germany; 3 Department of Diagnostic and Interventional Radiology, Charité Universitätsmedizin, Corporate Member of Freie Universität Berlin and Humboldt-Universität Zu Berlin, Campus Virchow-Klinikum (CVK), Berlin, Germany; 4 Department of Anaesthesiology and Intensive Care Medicine, University Hospital Leipzig, Leipzig, Germany

**Keywords:** Gamification, Medical learning, Chest radiography, Chest x-ray, Pneumothorax

## Abstract

**Background:**

Medical education could benefit from the introduction of novel teaching techniques. The present study aimed to assess the effect of a gamified mobile app (LuluRad) to improve pneumothorax detection on chest radiographs (CXRs).

**Methods:**

In this prospective, single-center randomized trial, third-year medical students were individually randomized using a coin toss to app-based learning (*n* = 60) or script-based learning (*n* = 66). Participants completed pre- and post-learning intervention CXR pneumothorax tests. The primary study outcome was pre/post diagnostic accuracy change between the app-based and script-based group. Secondary endpoints were changes in sensitivity and specificity.

**Results:**

In the app group, accuracy increased from 50.8% to 65.0% (*p* = 0.015) and sensitivity from 53.3% to 67.2% (*p* = 0.022), while specificity did not change significantly (*p* = 0.068). In the script group, accuracy did not significantly change (*p* = 0.132), sensitivity decreased from 64.1% to 41.9% (*p* < 0.001), and specificity increased from 39.4% to 51.0% (*p* = 0.006). Between-group change scores demonstrated greater increases in accuracy (Δ + 14.2 vs. − 5.3% points (pp); *p* < 0.001) and sensitivity (Δ + 13.9 vs. − 22.2 pp; *p* < 0.001) in the app group, while specificity did not change significantly between groups (*p* = 0.378).

**Conclusion:**

App-based learning was associated with greater increases in pneumothorax detection accuracy compared with conventional self-study among third-year medical students. Given the study design, these findings should be considered exploratory. While confirmation in larger studies is required, this approach appears promising and may represent a valuable adjunct to traditional teaching.

**Trial registration:**

This trial was not prospectively registered due to its educational study design and the absence of any potential harm or disadvantage for participants.

**Supplementary Information:**

The online version contains supplementary material available at 10.1186/s12909-026-09167-x.

## Background

Chest radiograph (CXR) interpretation is a fundamental skill for physicians, yet it remains challenging and error-prone even for experienced radiologists [1]. This challenge is even greater for trainees, as radiograph interpretation skills are often suboptimal among medical students and junior doctors [2, 3]. Among the various CXR pathologies, pneumothorax is one of the most important conditions to identify, as delayed recognition can be life-threatening [4].

Despite this need, medical school curricula often devote very limited time to radiology training [5]. As a consequence, many students reach clinical rotations with little exposure and low confidence in interpreting imaging studies [6]. This educational gap can carry over into residency, contributing to continued high error rates in imaging interpretation among junior doctors [2, 3]. Conventional teaching methods alone may not adequately equip medical students with the diagnostic imaging skills they need.

In response, educators are increasingly turning to e-learning and interactive training tools to augment traditional radiology education. Digital learning modules offer a number of advantages. They allow students to practice image interpretation in a self-paced and low-pressure environment. Such interactive, case-based learning has been shown to foster active engagement and improve skill acquisition [7]. Indeed, several studies have reported that e-learning modules for CXR interpretation are associated with improvements in students’ diagnostic accuracy and confidence, although the available evidence is heterogeneous and often lacks direct comparisons with conventional teaching [8, 9]. A recent systematic review summarized the evidence on e-learning in radiological image interpretation and found that digital interventions are generally associated with improved knowledge and diagnostic performance, despite heterogeneity in study design [10]. However, integration of structured e-learning into radiology curricula remains inconsistent, potentially due to limited curricular time and infrastructural constraints. These factors highlight the need for pragmatic, easily deployable digital tools that complement traditional teaching.

Beyond standard e-learning, gamification has emerged as a promising strategy to further increase learner engagement in medical education. Gamification refers to incorporating game-design elements, such as scoring points, level progression, timed challenges, and rewards into educational activities. Evidence suggests that gamified learning experiences not only boost students’ motivation and participation, but also improve their knowledge retention and skill acquisition [11]. The field of radiology, with its visual and problem-solving nature, is particularly well suited for gamified learning [12].

Given this background, we sought to investigate whether a mobile gamified app could enhance medical students’ CXR interpretation performance compared with conventional study methods. We developed and evaluated a publicly available smartphone app (LuluRad) for Android and iOS that functions as an interactive, gamified CXR training tool specifically designed to improve medical students’ ability to detect pneumothorax on CXR.

Although interactive e-learning modules can improve medical students’ CXR interpretation skills and confidence, the available evidence is largely derived from web-based interventions and often lacks direct comparisons with conventional teaching [8, 9, 13]. Few studies have evaluated a publicly available smartphone app that can be readily integrated into routine undergraduate teaching without institutional software infrastructure. To address this gap, we conducted a prospective, single-center randomized trial, comparing app-based learning with script-based self-study using objective pre/post changes in accuracy, sensitivity and specificity as endpoints.

## Methods

### Study design

This prospective, single-center randomized trial investigated the diagnostic performance of medical students in detecting pneumothorax on CXR. Participants were third-year medical students of the Medical Faculty, Leipzig University, Leipzig, Germany and recruited during regular teaching seminars (10 seminars, 12–14 students each). Third-year medical students were selected because, within our curriculum, they have already completed foundational anatomy training and introductory lectures on the technical principles of radiography, resulting in a relatively homogeneous baseline level of prior knowledge. Participants were recruited between March and November 2025. Inclusion criteria were voluntary participation and attendance at the scheduled teaching seminars. The exclusion criterion was lack of informed consent. Each student was individually randomized to the app-based or script-based learning group using a coin-toss procedure. The randomization was performed by the investigators, without blocking or stratification. Both groups completed a CXR performance test before and after the learning intervention. No blinding was applied in this study.

Students were anonymized throughout the study. Each participant received an identification code to enable matching the data of the pre- and post-intervention test.

Primary study outcomes were pre/post diagnostic accuracy change, between the app-based and script-based group. Secondary endpoints were changes in sensitivity and specificity.

An a priori power analysis was conducted for between-group difference in change in accuracy from pre- to post-test. Assuming a moderate effect size (Cohen’s d = 0.6), a statistical power of 0.80, and a two-sided independent t-test with a significance level of α = 0.05, a total sample size of 90 participants was required to detect a statistically significant difference. To overcome the risk of drop outs and the potential for further subgroup analyses, the recruitment was planned to oversize the participants number with two study groups. A total of 126 students were included in the study (app group: *n* = 60; script group: *n* = 66). Although non-parametric tests were applied in the final analysis due to non-normality, the sample size was considered sufficient to detect clinically relevant group differences.

For demographics, the participants were asked for age, gender and previous radiology experiences of curricular and extracurricular teaching in radiology departments. Only anonymized data were used for analysis, and no direct identifiers were collected.

### Learning intervention (app/ script)

Students assigned to the app group used the LuluRad Pneumothorax mobile learning application. The app is publicly available for iOS devices via the Apple App Store (https://apps.apple.com/us/app/lulurad-pneumothorax/id6740251256) and for Android devices via the Google Play Store (https://play.google.com/store/apps/details? id=com.lulurad.lulurad&hl=de). Participants were instructed to download and use the application independently during the study period. The app presents 20 anonymized CXRs and requires learners to decide whether a pneumothorax is present or not.

**Fig. 1:**
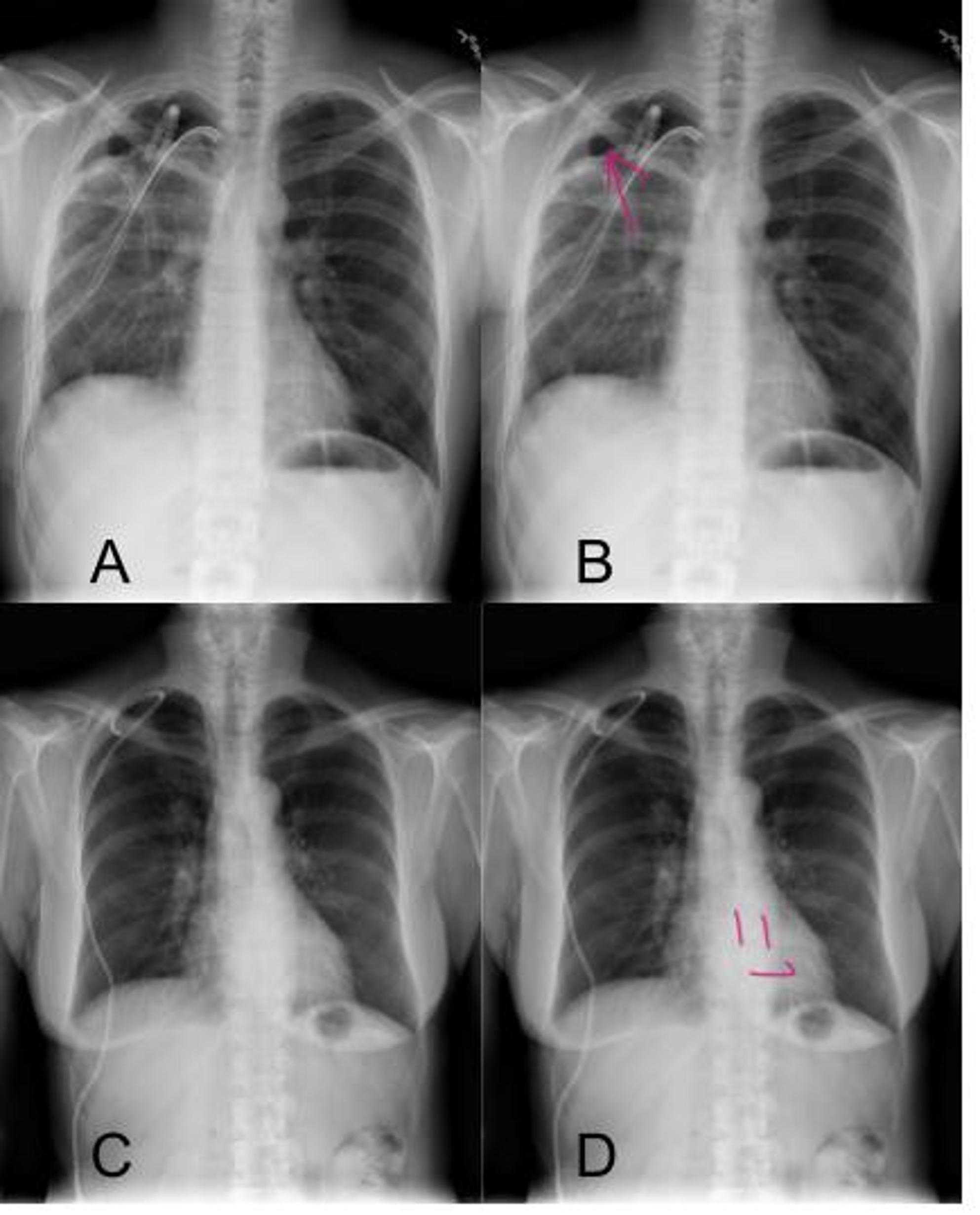
Representative chest radiograph (CXR) cases from the app. **A** Example of a CXR showing a pneumothorax in the right lung apex. **B** Instant feedback provided by the app, with the pneumothorax margin marked by a red arrow. **C** Example of a CXR without pneumothorax. **D** Instant feedback indicating a CXR without pneumothorax, illustrated by a smiley marker over the cardiac silhouette

After each decision, instant feedback is provided: a correct answer triggers a green highlight and a positive sound, whereas an incorrect answer results in a red highlight and a negative sound. Additionally, in pneumothorax cases, the pneumothorax margin is marked with a red arrow. In cases without pneumothorax a smiley icon appears over the cardiac silhouette. A continuous score counter is displayed throughout, and a summary score is shown upon completion. Representative examples of CXRs used in the app are shown in Fig. 1. An overview of the LuluRad mobile application interface and its feedback mechanisms is provided in Supplementary Material S1.

The control group received a printed educational script containing an introduction to pneumothorax, clinical background and current treatment guidelines, anatomical and radiographic fundamentals, a detailed description of pneumothorax signs on CXR, and three illustrative pneumothorax case examples. An English version of the educational script is provided in Supplementary Material S2.

All radiographs used in the app and script originated from publicly available, anonymized datasets approved for educational, clinical, scientific, and commercial use [14]. Students were instructed to review the material independently at home.

### Assessment of the learning effect

Testing was conducted in two sessions. The test radiographs were selected from an institutional teaching collection. A total of 12 cases (six with pneumothorax and six without pneumothorax) were independently reviewed by two radiologists with 4 and 8 years of experience, and only cases with unanimous agreement were included as the reference standard. Three pneumothorax cases and three cases without pneumothorax were randomly assigned to the pre-learning intervention test (pre-test), and the remaining cases to the post-learning intervention test (post-test), resulting in a balanced distribution between both tests. Students were not informed about the proportion of normal and abnormal cases in advance. At the beginning of the first seminar, students completed the pre-test. They were instructed to provide a binary response (pneumothorax present or absent). Following the pre-test, students were instructed to perform the learning intervention (app or script) at home. The content of the seminar was unrelated to the test subject and dealt with bone imaging. Two days later, the students attended the second seminar and completed the post-test with six different chest radiographs. All test cases were distinct from the learning intervention cases, as the pre- and post-test radiographs originated from our institutional teaching collection, while the app and script cases were derived from a publicly available CXR dataset [14]. In addition to the randomized trial cohort, an independent sample of 32 third-year medical students was recruited to assess potential differences in difficulty between the pre- and post-intervention test sets. These students did not participate in the randomized intervention and completed both six-case test sets consecutively in a single session without any educational intervention. The pre- and post-learning intervention chest radiograph test sets are provided in Supplementary Material S3.

### Ethical considerations

A formal ethics approval was waived by the Ethics Committee of the Medical Faculty, Leipzig University, Leipzig, Germany, as this was an anonymized educational intervention study with voluntary participation and no potential for harm or negative effect for the final exam of radiology. The study was conducted in accordance with the ethical principles of the Declaration of Helsinki. All participants provided informed consent prior to inclusion.

### Statistical Analysis

Statistical analysis was performed using IBM SPSS Statistics (Version 29, SPSS Inc., Chicago, Illinois, USA). All randomized participants were analysed according to their assigned study group. No participants were excluded from the analysis due to missing data. Baseline group differences were assessed using independent samples t-tests for continuous variables and χ² tests for categorical variables. Normality of the distribution of performance metrics and change scores was assessed using Shapiro–Wilk tests, which indicated significant deviations from normality (*p* < 0.001). Therefore, non-parametric tests were applied. Within-group comparisons between pre-learning intervention test and post-learning intervention test performance were performed using the Wilcoxon test. Between-group comparisons of performance change (Δ values) were analyzed using the Mann–Whitney U test. Statistical significance was set at α = 0.05 (two-tailed). Effect sizes for non-parametric comparisons were calculated as r.

## Results

### Participant characteristics

A total of 126 medical students were included (app: *n* = 60; script: *n* = 66). No participants were excluded, as all eligible students provided informed consent. A detailed description of the demographics of the groups is provided in Table 1. In short, the groups did not differ significantly in age (mean years app: 22.37; script: 22.41, *p* = 0.888), gender (app: 83.3% female; script: 80.3% female, *p* = 0.660) and previous radiology experience (app: 0.0%; script: 1.5%, *p* = 1.000).

**Table 1:** Participant characteristics and training

	App (n = 60)	Script (n = 66)
Age (years)	22.37 (± 1.340)	22.41 (± 1.945)
	p = 0.888	
Gender	83.3% female	80.3% female
	p = 0.660	
Previous radiology experience*	0%	1.5% (n = 1)
	p = 1.000	
Time spent learning	5.68 min (± 1.76)	4.65 min (± 2.99)
	p = 0.019	
Usage alone vs. in group	69% alone	100% alone
	p < 0.001	

Values are presented as mean ± standard deviation or percentage, as appropriate

p-values indicate between-group comparisons (independent t-test or χ² test). *One student had completed training as a radiologic technologist

Students in the app group spent significantly more time on the learning intervention compared with the script group (5.68 min vs. 4.65 min, *p* = 0.019). Furthermore, learning modality differed between groups. While all students in the script group worked individually, 31% of students in the app group used the tool collaboratively (*p* < 0.001).

### App group performance

Use of the learning app was associated with a significant improvement in pneumothorax diagnostic performance. Accuracy increased from 50.8% to 65.0% (*p* = 0.015). Sensitivity increased from 53.3% to 67.2% (*p* = 0.022). Specificity did not change significantly (*p* = 0.068).

Diagnostic performance before and after the learning intervention is summarized in Table 2.

**Table 2:** Diagnostic performance before and after the learning intervention

	App		Script	
pre Sensitivity	53.3% (± 25.4)	p = 0.022(r = 0.30)	64.1% (± 18.8)	p < 0.001(r = -0.51)
post Sensitivity	67.2% (± 40.0)		41.9% (± 33.7)	
pre Specificity	48.3% (± 30.3)	p = 0.068(r = 0.24)	39.4% (± 29.1)	p = 0.006(r = 0.34)
post Specificity	62.8% (± 25.4)		51.0% (± 22.0)	
pre Accuracy	50.8% (± 21.1)	p = 0.015(r = 0.31)	51.8% (± 17.6)	p = 0.132(r = -0.19)
post Accuracy	65.0% (± 29.1)		46.4% (± 20.8)	

Before (pre) and after (post) learning intervention test results for sensitivity, specificity and overall diagnostic accuracy are shown for the app and script groups. Values are presented as mean ± standard deviation. p-values refer to within-group comparisons (Wilcoxon test). Effect sizes are reported as r

### Script group performance

Different trends were observed in the script-based learning group. Accuracy did not change significantly (*p* = 0.132). Sensitivity decreased from 64.1% to 41.9% (*p* < 0.001). Specificity increased from 39.4% to 51.0% (*p* = 0.006).

### Script and app performance change comparison

Change scores demonstrated a significant advantage of the app group over the script group. ΔAccuracy was significantly higher in the app group compared with the script group (14.2 vs. -5.3% points (pp), *p* < 0.001). Also ΔSensitivity was significantly higher in the app group compared to the script group (13.9 vs. -22.2 pp; *p* < 0.001). ΔSpecificity did not differ significantly between groups (*p* = 0.378).

Between-group comparisons of performance change are shown in Table 3.

**Table 3:** Comparison of performance change between the app and script group

	App	Script
Δ Sensitivity	13.9 pp (± 43.5)	-22.2 pp (± 37.1)
	p < 0.001 (r = 0.41)	
Δ Specificity	14.4 pp (± 46.1)	11.6 pp (± 35.8)
	p = 0.378 (r = 0.08)	
Δ Accuracy	14.2 pp (± 39.3)	-5.3 pp (± 25.7)
	p < 0.001 (r = 0.31)	

Performance change (Δ) before and after learning intervention for sensitivity, specificity and overall diagnostic accuracy are shown for the app and script groups. Δ values are presented as percentage points (pp) ± standard deviation. p-values indicate between-group comparisons (Mann-Whitney U test). Effect sizes are reported as r

### Comparison of pre- and post-test sets

In an independent sample of 32 students completing both test sets without learning intervention, accuracy was lower in the post-test compared to the pre-test (52.6% vs. 45.3%, *p* = 0.043). Also sensitivity was reduced in the post-test (59.4% vs. 45.8%, *p* = 0.030), whereas specificity did not differ significantly (*p* = 0.870).

The comparison of pre- and post-test sets performance are shown in Table 4.

**Table 4:** Comparison of pre- and post-intervention test sets in an independent sample.

	pre-test	post-test	p-value
Sensitivity	59.4% (± 16.4)	45.8% (± 31.4)	p = 0.03 (r = -0.38)
Specificity	45.8% (± 30.2)	44.8% (± 23.4)	p = 0.87 (r = 0.03)
Accuracy	52.6% (± 14.1)	45.3% (± 17.5)	p = 0.043 (r = -0.36)

All participants completed both test sets in a single session without prior intervention (n = 32). Values are presented as mean ± standard deviation. p-values refer to paired comparisons (Wilcoxon test). Effect sizes are reported as r

## Discussion

The results of this randomized trial indicate a significant advantage of gamified app-based learning over conventional script-based self-study. Between-group comparisons of change scores showed significantly greater increases in the app group for accuracy (Δ + 14.2 vs. −5.3% points (pp); *p* < 0.001) and sensitivity (Δ + 13.9 vs. −22.2 pp; *p* < 0.001), whereas changes in specificity did not differ significantly between groups. Within-group analyses further demonstrated that students who trained with the app (LuluRad) achieved a significant increase in pneumothorax detection sensitivity (53.3% to 67.2%) and overall diagnostic accuracy (50.8% to 65.0%). In contrast, the script group showed no significant change in overall accuracy, while sensitivity decreased (64.1% to 41.9%). An additional analysis in an independent cohort demonstrated that the post-test set was intrinsically more difficult, with significantly lower sensitivity and overall accuracy compared to the pre-test. Therefore, the decline observed in the script group is likely at least partially attributable to differences in test difficulty rather than a true deterioration in performance following script-based learning. Baseline sensitivity was lower in the app group (53.3%) compared to the script group (64.1%). While regression to the mean cannot be fully excluded, the direction of changes is consistent with a potential intervention effect. Although the improvement in the app group despite the higher intrinsic difficulty of the post-test is encouraging, this imbalance complicates interpretation of both within- and between-group changes. The magnitude of observed changes cannot be directly compared to results expected under equivalent test difficulty. In addition, the independent cohort pre-/post-test analysis is based on a small number of cases (*n* = 32). Therefore, the findings should be interpreted cautiously and as exploratory.

Our findings align with an emerging body of literature on gamified radiology education. A study using a web-based training game (RapRad) reduced the error rate from 39% to 22% after completion of the game curriculum [13]. However, no control group for comparison was used in this study. Similarly, another single-armed study reported that the use of an interactive web-based e-learning module for CXR interpretation led to a significant improvement in overall performance, increasing from 13.2 ± 3.36 to 15.8 ± 3.40 out of 25 [8]. A recent quasi-experimental study found that gamified training combined with an in-person rotation into clinical radiology led to greater improvements in CXR interpretation skills than rotation alone, measured as Multiple-Choice Questions (MCQ) knowledge score changes and end-of-course Objective Structured Clinical Examination (OSCE) skills scores [15]. The present study corroborates previous findings and extends the evidence base by employing a randomized trial design.

By demonstrating objective increases in both sensitivity and overall accuracy with app-based learning, our study extends these prior reports and adds evidence that gamification can translate into measurable improvements in diagnostic performance. The greatest improvement we observed was in sensitivity. This is clinically important because failing to detect a pneumothorax on a CXR can have life-threatening consequences. These findings suggest that an active learning tool such as LuluRad may be valuable for training CXR interpretation.

The superior performance observed with the gamified app may be explained by several learning principles. The app promotes active cognitive engagement by requiring learners to interpret each case and commit to a decision, rather than relying on passive exposure to written descriptions. Active participation has been associated with better skill retention [16]. In our study, app users practiced across twenty varied clinical cases and received immediate feedback, which may have supported more efficient pattern recognition and consolidation of key radiographic features of pneumothorax. Overall, this aligns with experiential, feedback-driven learning approaches that are particularly well suited to visual, problem-solving tasks such as image interpretation [17]. Moreover, the app’s self-paced, on-demand format aligns with current approaches to self-directed learning in medical education [18]. Notably, approximately one-third of participants in the app group chose to learn collaboratively, despite the intervention being assigned as individual home study. This unplanned peer learning indicates the app not only engaged students individually but also stimulated interactive discussion, that the written script lacked.

Despite the encouraging results, our study has several limitations to address. First, it was conducted at a single institution with a specific cohort of third-year medical students, which may limit generalizability. Second, our assessment of pneumothorax detection was immediate and short-term. It remains unclear whether the increases in accuracy and sensitivity persist over time. Third, the app combined interactivity, feedback, and gamified features, whereas the script provided more extensive explanatory content but little hands-on practice. Thus, our study design cannot isolate which specific element of the app was most responsible for the performance improvement. Also, the script intervention may have promoted a different type of learning, which cannot be distinguished by a binary accuracy measure. Fourth, engagement with the learning intervention was brief overall, with students spending approximately five minutes on the assigned material. This is shorter than the time medical students would typically spend with a learning resource in practice and limits conclusions about sustained educational impact [19]. Moreover, students in the app group spent more time than those in the script group and partly used the app collaboratively, which may have contributed to their superior performance independently of gamification. Fifth, our outcome measures focused on diagnostic accuracy within a controlled test setting. Whether training with the app translates into better results for the final radiology exam or even later clinical performance, was beyond the scope of this study. Sixth, a limitation is the skewed gender distribution, with a predominance of female participants in both groups. Nevertheless, this mirrors the current gender distribution among medical students in Germany, partially mitigating concerns regarding generalizability. Seventh, the small number of test cases per participant substantially limited the precision of the performance metrics. Because each student evaluated only three pneumothorax-positive and three pneumothorax-negative CXR per test session, sensitivity and specificity could each take only four possible values (0%, 33.3%, 66.7%, or 100%). Accordingly, a single additional correct or incorrect answer could change these metrics by 33.3% points. Accuracy, based on six cases, likewise changed in steps of 16.7% points. Eighth, the post-test was intrinsically more difficult than the pre-test, as demonstrated in an independent cohort. This imbalance may have biased results toward lower post-test performance. However, the improvement in the app group occurred despite the more difficult post-test, supporting a true intervention effect. Another limitation is the binary assessment format, which does not allow differentiation between true diagnostic reasoning and correct guessing, particularly given the small number of test cases. In addition, the similarity between the app’s decision-based format and the final assessment may have introduced a testing effect, potentially favoring the app group. Finally, several CONSORT criteria were not fully met due to the educational study design, including the absence of blinding and allocation concealment. Nevertheless, outcomes were objectively assessed, and no relevant baseline differences between groups were observed. These limitations temper our conclusions and highlight areas for further investigation.

In conclusion, gamified app-based learning was associated with greater increases in pneumothorax detection accuracy compared with conventional self-study among third-year medical students. Given the methodological constraints, the present results should be considered exploratory. While further validation in larger studies is required, integrating short, case-based digital modules into radiology teaching may help strengthen early competency in CXR interpretation.

## Supplementary Information

Supplementary Material 1.

Supplementary Material 2.

Supplementary Material 3.

## Data Availability

The datasets generated and analyzed during the current study are available from the corresponding author on reasonable request.

## Abbreviations

CONSORT: Consolidated Standards of Reporting Trials
CXR: Chest radiograph (chest X–ray)
MCQ: Multiple–choice questions
OSCE: Objective Structured Clinical Examination
