# Supplementary material for: Randomized comparison of gamified mobile app–based training versus conventional learning for pneumothorax detection in chest radiographs
Source: BMC Med Educ. 2026 Apr 13;26:637. doi: 10.1186/s12909-026-09167-x (PMC13091258; doi:10.1186/s12909-026-09167-x)

**Supplementary Material 1. Overview of the LuluRad mobile application interface and feedback mechanisms.**
(A) Start screen of the LuluRad app. (B) Example case presentation with a chest radiograph (CXR) and binary decision options “Kein Pneu” (No pneumothorax) and “Pneu” (Pneumothorax); additional interface terms include “Fall” (Case) and “Punkte” (Points). (C) Zoomed in view of the same case. (D) Immediate feedback after a correct pneumothorax diagnosis, with the pneumothorax margin marked by a red arrow and the correct answer highlighted in green. (E) Example of a CXR without pneumothorax prior to answer selection. (F) Zoomed in view of the same case. (G) Immediate feedback after an incorrect pneumothorax response. A smiley face displayed over the cardiac silhouette represents the absence of pneumothorax. The incorrect answer is highlighted in red to indicate error. (H) Final results screen summarizing user performance after completion of all cases, including “Herzlichen Glückwunsch!” (Congratulations!), “Du hast 13 von 20 Fälle richtig gelöst” (You solved 13 out of 20 cases correctly), and “Hauptmenü” (Main menu).
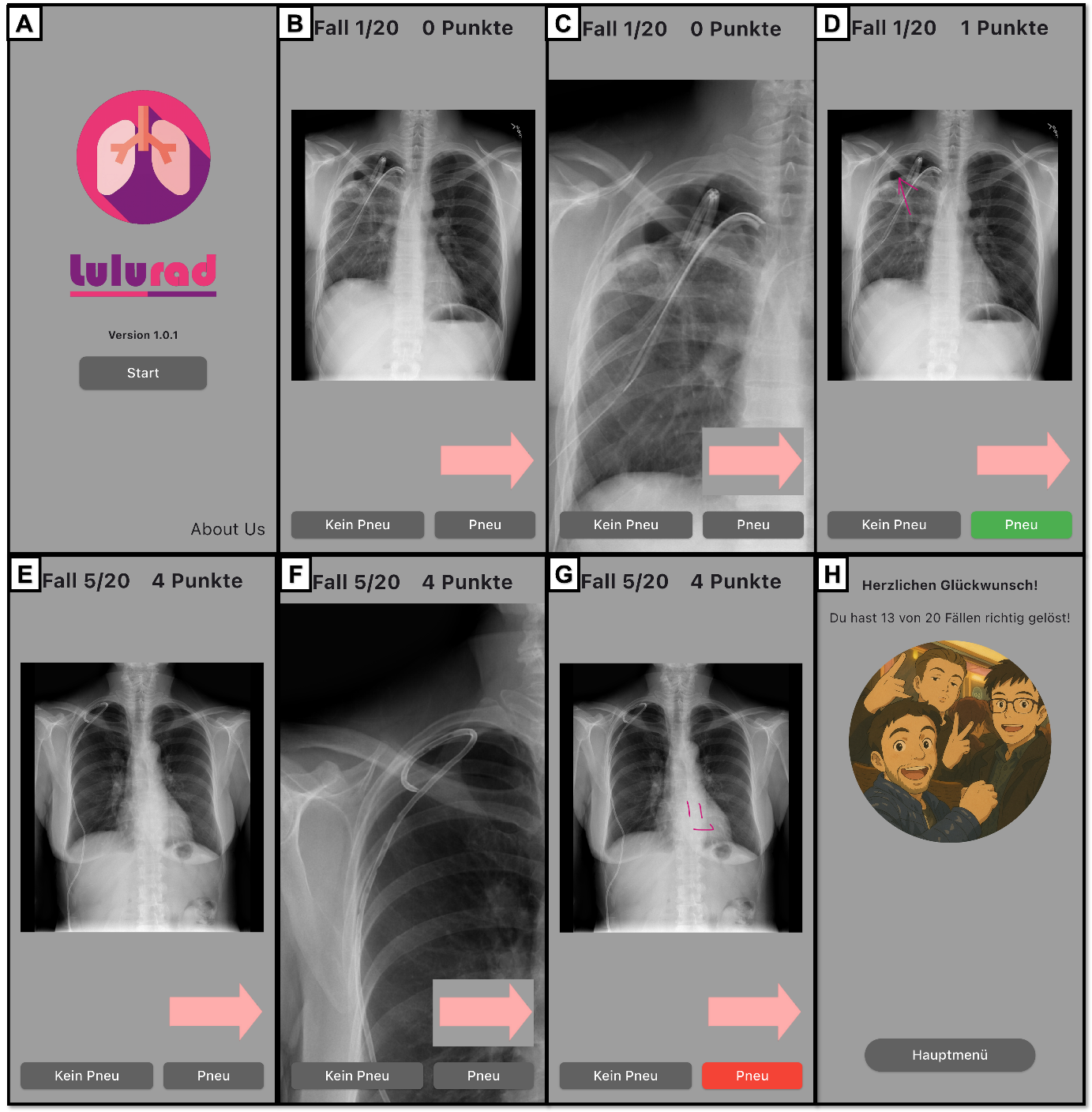

Supplement: Supplementary file 1 — Supplementary Material 1. [file 12909_2026_9167_MOESM1_ESM.docx]
