# Supplementary material for: Randomized comparison of gamified mobile app–based training versus conventional learning for pneumothorax detection in chest radiographs
Source: BMC Med Educ. 2026 Apr 13;26:637. doi: 10.1186/s12909-026-09167-x (PMC13091258; doi:10.1186/s12909-026-09167-x)
Supplement: Supplementary file 2 — Supplementary Material 2. [file 12909_2026_9167_MOESM2_ESM.docx]

**Supplementary Material S2. English Translation of the Pneumothorax Teaching Script**

**1. Introduction**

A pneumothorax is a collapse of the lung caused by air entering the pleural space. Under normal conditions, negative pressure within the pleural cavity keeps the lung expanded.

The most common causes are:

- Spontaneous (primary/idiopathic or secondary, e.g., due to COPD)
- Traumatic
- Iatrogenic (e.g., after central venous catheter placement)

**2. Clinical Presentation**

Typical symptoms include sudden onset, usually unilateral chest pain and dyspnea.

In the case of a tension pneumothorax, additional findings may include:

- Hypotension
- Cyanosis
- Jugular venous distension
- Possible circulatory collapse

**3. Radiographic Diagnosis According to the German S3 Guideline (2018)**

For the initial diagnosis of pneumothorax, an upright posteroanterior (p.a.) chest radiograph taken at maximum inspiration is recommended. It is the imaging modality of first choice for both primary and secondary pneumothorax.

Diagnostic signs:

- Visible displacement of the visceral pleural line
- Absence of peripheral pulmonary vascular markings
- Possible presence of subcutaneous emphysema

Note: Precise estimation of the size of a pneumothorax is difficult, particularly in patients with bullous or cystic lung disease. In such cases, CT imaging should be considered depending on clinical relevance.

**4. Additional Radiographic Techniques According to the S3 Guideline**

- Lateral chest radiograph: Helpful in approximately 14% of cases, especially in patients with pre-existing lung disease (e.g., COPD or pulmonary fibrosis) and secondary spontaneous pneumothorax. In primary spontaneous pneumothorax, the lateral view is usually unnecessary.
- Expiratory p.a. view: Not recommended, as it does not provide additional diagnostic benefit compared to inspiratory imaging.
- Supine chest radiograph (a.p. or lateral): Lower sensitivity than upright p.a. views. In intensive care patients, CT or ultrasound may be required for clarification.
  - A subpulmonic pneumothorax may present with a widened costophrenic recess (*deep sulcus sign*).
  - Ventral pneumothoraces are more difficult to detect and may appear as increased contour visibility of the mediastinum or heart.

Figure: Chest radiograph of a patient with pneumothorax*


This image shows a left-sided pneumothorax with the typical radiographic signs on an inspiratory p.a. chest radiograph:

- Visible visceral pleural line
- Absence of lung markings peripheral to the line
- Increased radiolucency in the affected area

**5. Treatment**

Management depends on severity and may include:

- Conservative management (observation)
- Chest tube drainage
- Surgical intervention

Recurrent primary pneumothorax should be treated surgically, usually via video-assisted thoracoscopic surgery (VATS).

Follow-up is typically performed in an outpatient setting. Air travel and high-altitude exposure should be avoided for approximately four weeks. Diving requires evaluation and clearance by a specialist in diving medicine.

**
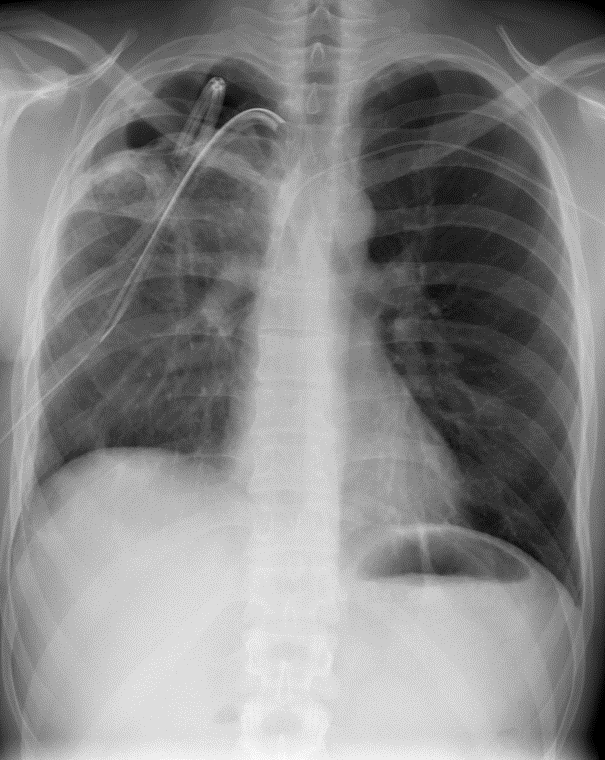
Case 1***

The chest radiograph shows a patient with two apically positioned chest tubes on the right side. A visible visceral pleural line is seen in the right apex with absence of peripheral lung markings, consistent with a pneumothorax.

**Case 2
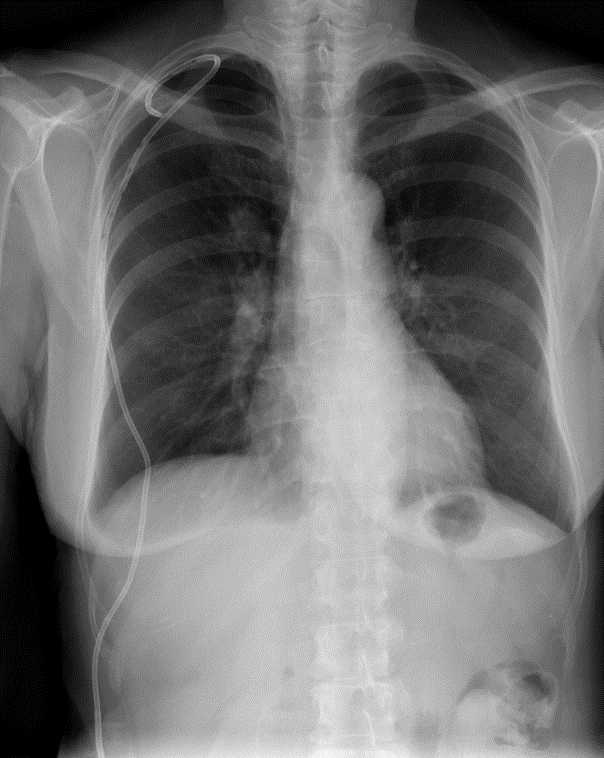
***

A right-sided chest tube is in place with apical positioning. There is no visible visceral pleural line, and pulmonary vascular markings extend to the periphery bilaterally. Therefore, there is no evidence of pneumothorax.

**Case 3***

A subtle visceral pleural line is visible laterally on the right side, with absence of peripheral pulmonary vascular markings beyond this line.
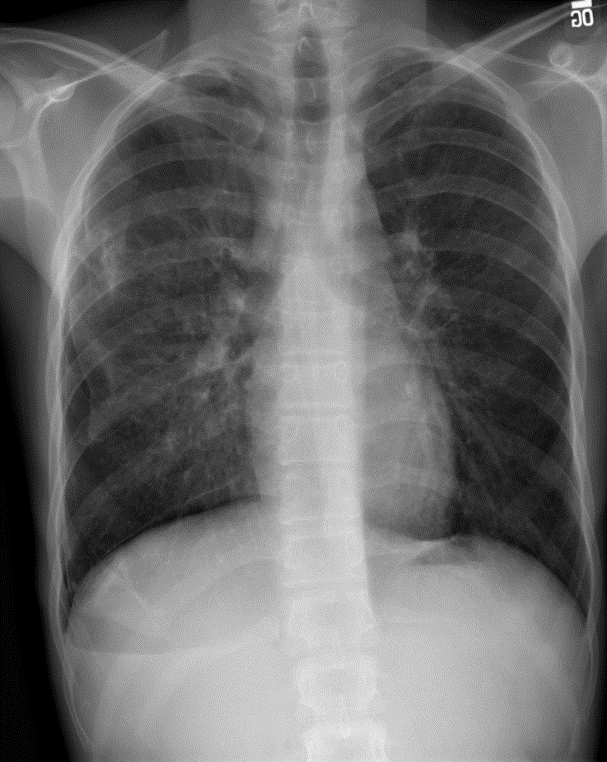
 Medially adjacent to the pleural line, there are linear opacities consistent with dystelectasis.

* All CXR used in this script were obtained from a publicly available dataset:

Wang X, Peng Y, Lu L, Lu Z, Bagheri M, Summers RM. *ChestX-Ray8: Hospital-Scale Chest X-Ray Database and Benchmarks on Weakly-Supervised Classification and Localization of Common Thorax Diseases.* Proceedings of the IEEE Conference on Computer Vision and Pattern Recognition (CVPR). 2017; 3462–3471. doi:10.1109/CVPR.2017.369.
