# Supplementary material for: Randomized comparison of gamified mobile app–based training versus conventional learning for pneumothorax detection in chest radiographs
Source: BMC Med Educ. 2026 Apr 13;26:637. doi: 10.1186/s12909-026-09167-x (PMC13091258; doi:10.1186/s12909-026-09167-x)

**Supplementary Material S3. Pre- and Post-Learning Intervention Chest Radiograph Test Sets (A–L)**

A–F: Pre-learning intervention test set (6 cases: 3 with pneumothorax, 3 without pneumothorax). A: Right-sided pneumothorax. B: No pneumothorax. C: Left-sided pneumothorax. D: No pneumothorax. E: No pneumothorax. F: Right-sided pneumothorax.

G–L: Post-learning intervention test set (6 cases: 3 with pneumothorax, 3 without pneumothorax). G: Right-sided pneumothorax. H: No pneumothorax. I: No pneumothorax. J: No pneumothorax. K: Right-sided pneumothorax. L: Left-sided pneumothorax.


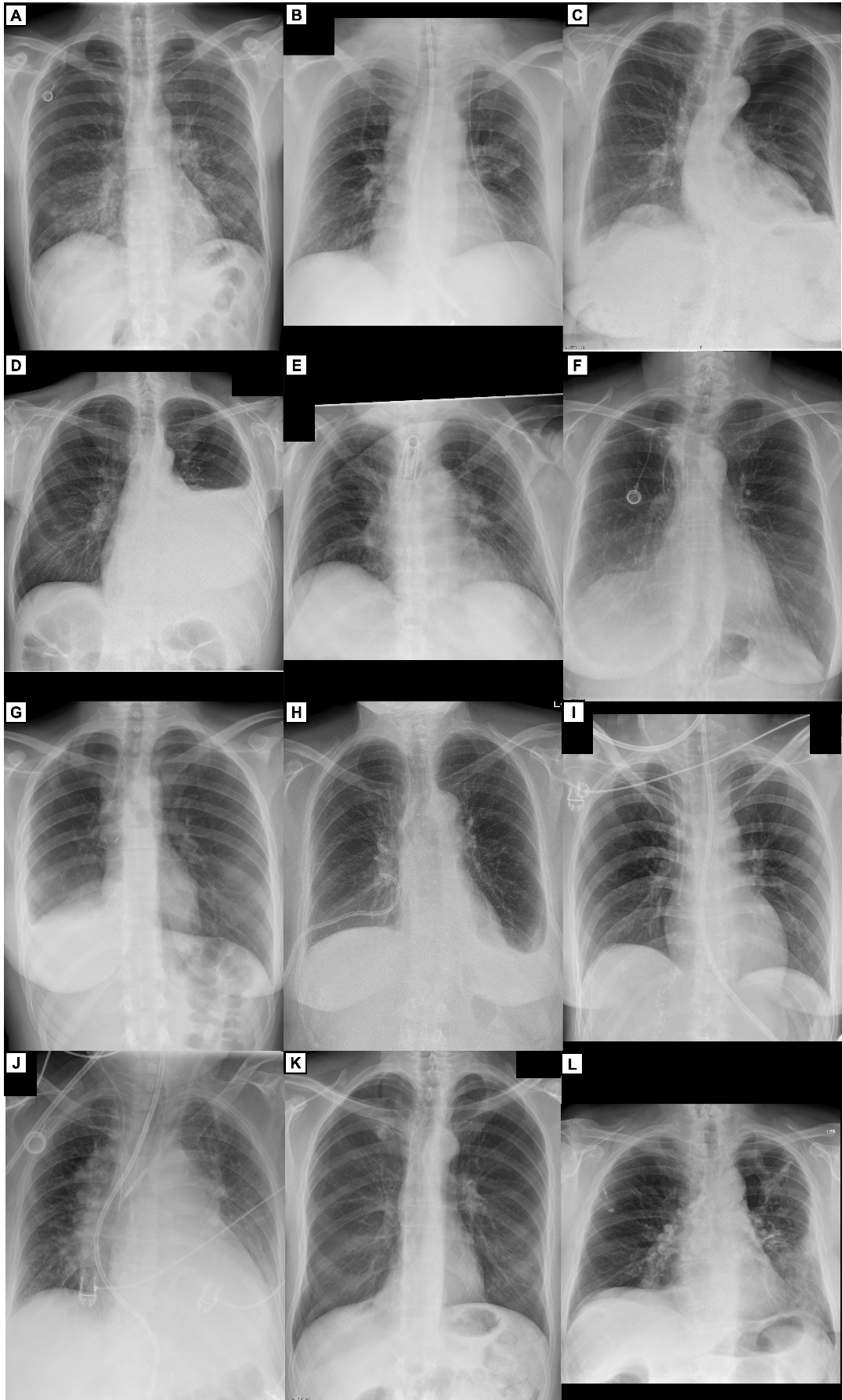

Supplement: Supplementary file 3 — Supplementary Material 3. [file 12909_2026_9167_MOESM3_ESM.docx]
